# Comparison of Neuroendoscopic and Microscopic Surgery for Unilateral Hemilaminectomy: Experience of a Single Institution

**DOI:** 10.3389/fsurg.2022.823770

**Published:** 2022-03-29

**Authors:** Wei Zeng, Haixiao Jiang, Shiwei He, Yukun Zhang, Bo Yu, Hui Wang, Cunzu Wang

**Affiliations:** ^1^Department of Clinical Medicine, School of Medicine, Yangzhou University, Yangzhou, China; ^2^Department of Clinical Medicine, Dalian Medical University, Dalian, China; ^3^Department of Neurosurgery, Clinical Medical College of Yangzhou University, Yangzhou, China

**Keywords:** endoscope, unilateral hemilaminectomy, intraspinal tumor, microscope, retrospective single center analysis

## Abstract

**Objective:**

This study was designed to compare the safety and efficacy of unilateral hemilaminectomy conducted under complete neuroendoscopic visualization (UHNV) relative to unilateral hemilaminectomy under total microscopic visualization (UHMV) for the treatment of patients diagnosed with intraspinal tumors.

**Methods:**

In total, 41 patients undergoing intraspinal tumor resection at Northern Jiangsu People's Hospital were included in this study, including 20 and 21 patients in the UHNV and UHMV groups, respectively. Intraoperative parameters including incision length, operative duration, number of vertebral laminae removed and intraoperative blood loss, as well as indicators of curative efficacy such as total tumor resection rates and postoperative symptom improvement rates, and safety indicators including complication rates, recurrence rates, spinal deformity rates, spinal instability incidence, and length of stay (LOS), were compared between the two groups.

**Results:**

In contrast to the UHMV group, patients in the UHNV group had a significantly shorter incision length and decreased intraoperative blood loss (*P* < 0.05), while the operative duration (*P* > 0.05) showed no statistical difference. Although the postoperative improvement and total tumor resection rates were enhanced, the difference was not statistically significant (*P* > 0.05). In comparison, the bedridden time and length of stay (LOS) were significantly shortened (*P* < 0.05) in the UHNV group. However, there were no significant differences in recurrence, incidence of complications, spinal deformity, and spinal instability (*P* > 0.05).

**Conclusion:**

Collectively, our findings indicate that UHNV is not inferior to the UHMV approach. Moreover, due to its safe and minimally invasive nature, UHNV represents a promising alternative to UHMV as a treatment for patients with intradural extramedullary tumors.

## Introduction

Only 15% of central nervous system tumors are intraspinal tumors, also called spinal cord tumors, which include intramedullary (around 5%) and extramedullary (approximately 95%) tumors that can occur in any part of the spinal canal (1). These are predominantly benign tumors that cause pain and various sensory, motor, reflex, and sphincter deficits (2). Surgical resection remains the most effective treatment option for the majority of intraspinal tumors (3). There are three commonly used surgical methods to date: total microscopic laminectomy, unilateral microscopic hemilaminectomy, and split microscopic laminotomy (4). Laminotomy is considered a secondary option due to its limited indications, which include dorsal midline intradural and extramedullary lesions, as well as the majority of intramedullary lesions. The studies by Weber et al., Lei et al., and Mobbs et al. (2, 5, 6). suggest that unilateral hemilaminectomy is safer and more effective than total laminectomy because it is minimally invasive and associated with a less negative impact on spinal stability. Furthermore, there is mounting evidence that unilateral hemilaminectomy should be the first-line clinical intervention (5–7). However, because only half of the vertebral laminae are removed during this procedure, the operating space is severely constrained, obstructing or obscuring microscopic visualization. Direct and adequate visualization is critical for safe dissection of tumors located ventrally, lesions that extend bilaterally in the spinal canal, and tumors densely adherent to the cord surface. In such cases, choosing a microscopic hemilaminectomy approach is inappropriate. As a result, microscopic hemilaminectomy is generally reserved for tumors with narrow width and situated on one side of the spine only (8). In contrast, the use of neuroendoscopy can offer superior visualization, overcoming the deficiencies associated with the microscopic approach (9). Therefore, its application value in minimally invasive skull base and pituitary tumor surgery is superior. However, whether this advantage can be demonstrated in spinal cord surgery is unknown. To that end, we compared the relative safety and efficacy of hemilaminectomy procedures performed to remove intraspinal tumors conducted under either microscopy or neuroendoscopy.

## Materials and Methods

### Patient Data

The present study was approved by the Ethics Committee of Northern Jiangsu People's Hospital (2022ky108). This study reviewed previous cases and included 41 intraspinal tumor patients who underwent treatment in the Department of Neurosurgery of Northern Jiangsu People's Hospital via a neuroendoscopic (*n* = 20) or microscopic (*n* = 21) approach from June 2018 to September 2020. Patients from June 2018 to June 2019 all received microscopic surgery, and the rest had surgery using the neuroendoscopic approach. Although our medical group consists of five doctors, all surgeries were performed by the same chief physician, while the other doctors offered assistance.

The inclusion criteria were as follows: (1) definite diagnosis of intraspinal tumors; and (2) surgical tumor resection using neuroendoscopic or microscopic hemilaminectomy. Exclusion criteria were as follows: (1) neuroendoscope-assisted microscopic surgery, and (2) patients lost to follow-up.

The present study assessed baseline data (age, sex, symptoms, and imaging findings associated with the upper and lower tumor segments, ventral or dorsal position, and position within the spinal canal), surgical approach (microscopic vs. endoscopic), and outcome indicators (incision length, number of vertebral laminae removed, operative duration, intraoperative blood loss, postoperative symptom improvement rates, tumor resection rates, complications, bedridden time, length of stay (LOS), recurrence rate, spinal deformity, and spinal instability (The estimate of spinal stability was based on the variations in sagittal and coronal curvature of the spine's surgical site by comparing preoperative X-ray or CT image data. An angular variation of less than 15 was considered good spinal stability; otherwise, it was defined as spinal instability)). Additionally, in schwannoma, postoperative limb numbness was defined as a postoperative sequela rather than a postoperative complication, while in meningioma, postoperative numbness at follow-up was defined as a postoperative complication to exclude the effects of surgery, anesthesia, and bed rest.

### Surgical Approach

**Figure 1:**
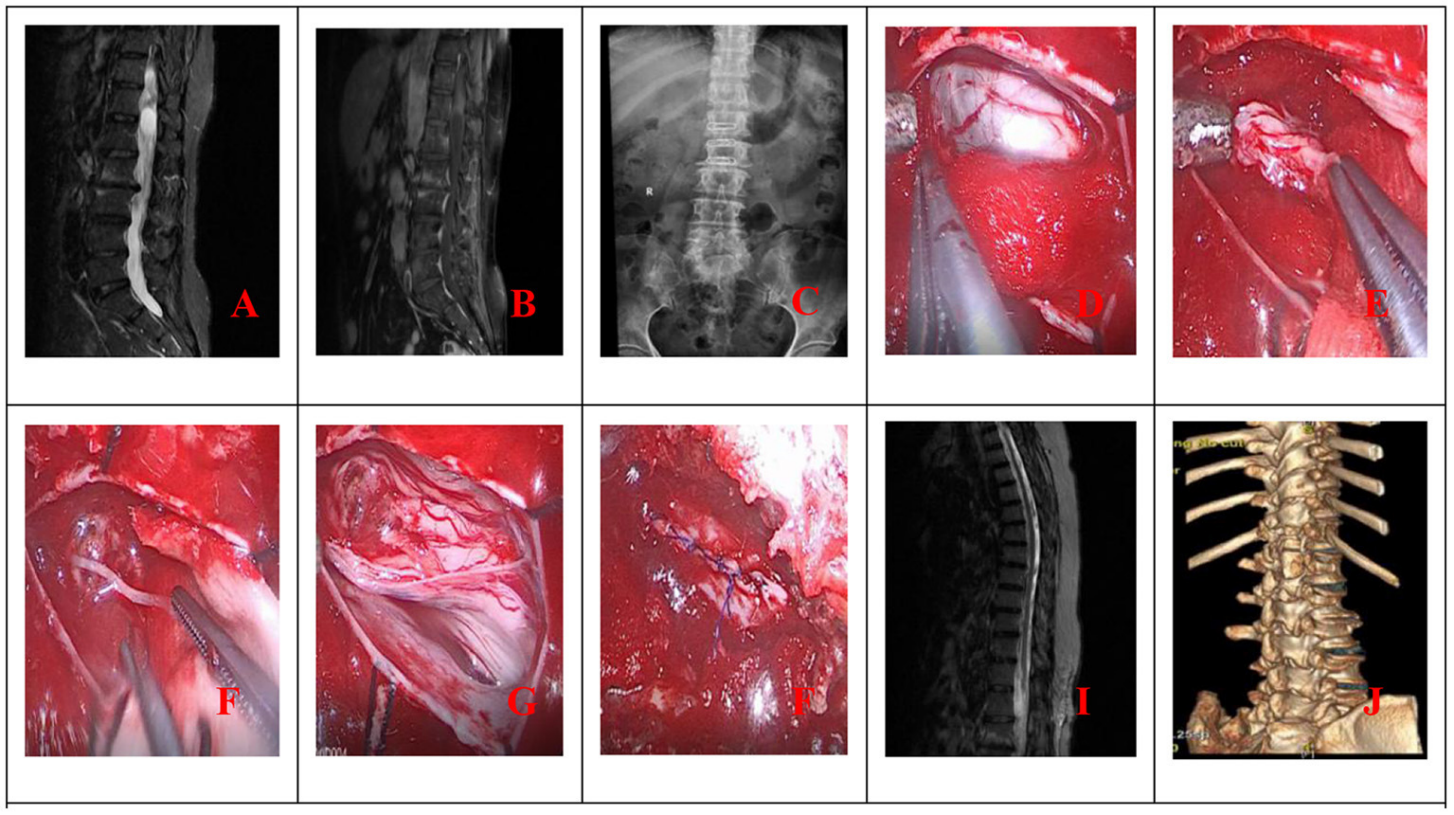
**(A)** Preoperative MRI; **(B)** Preoperative MRI enhancement; **(C)** Position the cone preoperatively; **(D)** Open the dura mater under endoscope; **(E)** Separate and pull out the tumor under endoscope; **(F)** Cut off the tumor-bearing nerve after endoscopic electrocoagulation and remove all the tumor; **(G)** Endoscope demonstrated good nerve root protection after tumor resection; **(H)** Continuous suture of spinal dura under endoscope; **(I)** Postoperative MRI revealed total tumor resection **(J)**. Postoperative 3D CT reconstruction.

**Figure 2:**
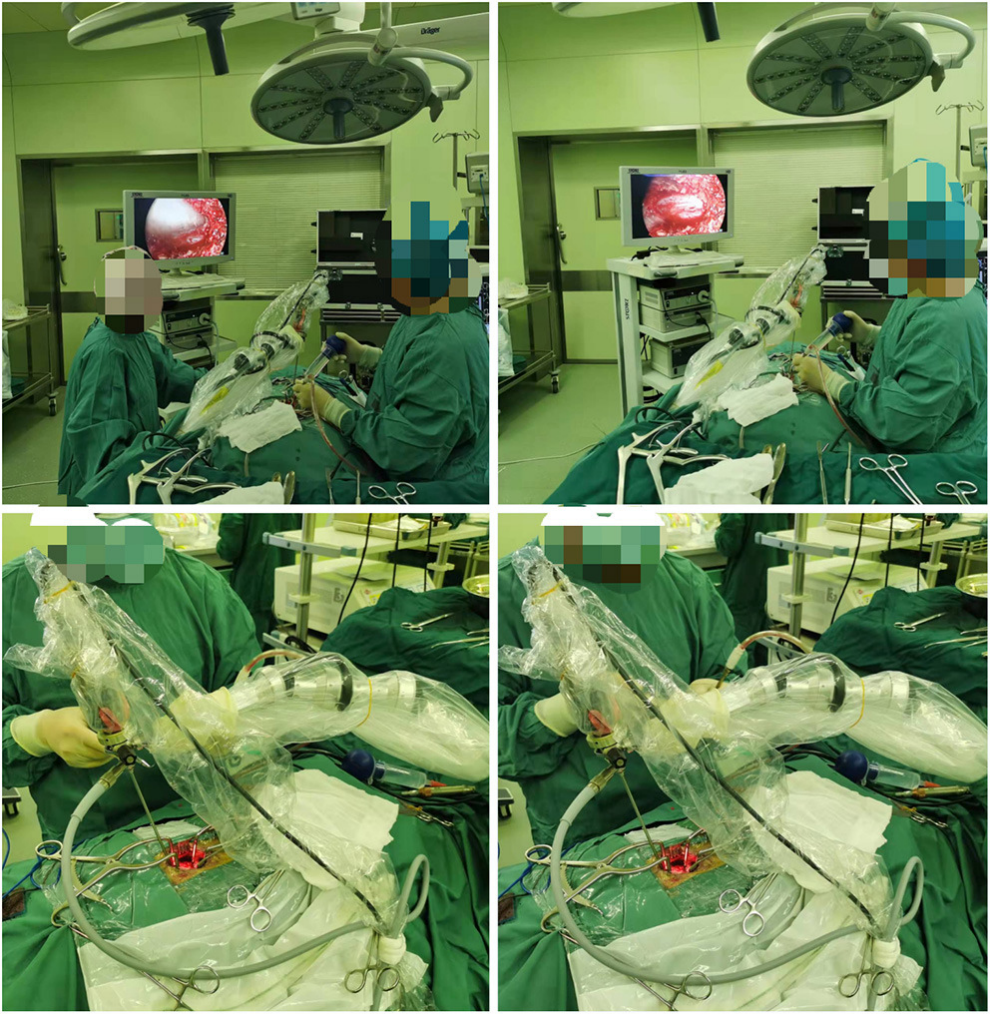
Describe the location of intraoperative neuroendoscopy.

The procedure for patients undergoing unilateral hemilaminectomy under complete neuroendoscopic visualization (UHNV) was performed using a Storz rigid neuroendoscope (4 mm in diameter, 0 or 30-degree lens), a pneumatic mechanical manipulator for endoscope fixation, and a high-definition imaging system. X-ray imaging was used to locate the vertebral segments affected by the intraspinal tumor. Patients adopted the prone position throughout the procedure, and the skin was cut along the posterior median line, with the length of the incision determined by the size of the tumor. The paravertebral muscles on the tumor side were cut and separated, leaving the spinous process and supraspinous ligaments intact, and the paravertebral muscle was manipulated to expose the location of the underlying tumor, creating a working channel for endoscopic visualization. Following neuroendoscopy fixed by a pneumatic mechanical manipulator, the corresponding vertebral laminae were partially removed under neuroendoscope according to location and size, and basal range of tumor, and the window size is suitable for the operation of two instruments such as an aspirator and a detacher under endoscopic visualization. (In the case of smaller lesions, an interlaminar partial hemilaminectomy of the two contiguous laminae was performed, similar to case 1). The endorhachis was cut longitudinally, and adhesions between the tumor envelope and the spinal cord as well as the nerve roots were carefully separated before resection to preserve the nerves and spinal cord. The position of the neuroendoscope was adjusted to avoid intraoperative blind spots caused by bony structures blocking the vertebral canal. Under neuroendoscopic guidance, the spinal dura mater was continuously sutured with non-injury sutures following tumor resection. To avoid the need for a drainage catheter, the paraspinal muscles were sutured to the interspinous ligaments (Figures 1, 2).

The surgical procedure for the UHMV group was the same as described above, but microscopic visualization was used instead.

### Postoperative Management and Follow-Up

Patients were encouraged to leave their beds and engage in functional exercise or other treatments as soon as possible following surgery. A computed tomography (CT) scan of the spinal canal was performed the day after surgery to assess the status of the vertebral laminae and detect any bleeding in the surgical site. Magnetic resonance imaging was used to assess tumor resection status 6 months after treatment (MRI). Annual MRIs and 3D reconstruction CT scans were then performed to assess outcomes such as tumor recurrence, spinal deformity, and spinal instability. All patients were followed up postoperatively via email, phone call, SMS, return visits, to name a few, to assess clinical outcomes such as symptom improvement, complications, and imaging findings.

### Statistical Analysis

Continuous data were expressed as means ± standard deviation (x ± s) and compared using Student's *t*-tests, whereas categorical data were expressed as rates (%) and compared using chi-squared tests and Fisher's exact test. A *P*-value < 0.05 was deemed statically significant. SPSS version 23.0 was used for all statistical analyses.

## Results

### Baseline Indicators in the Two Treatment Groups

There were no significant differences in baseline characteristics such as age, gender, or symptoms between patients in the two treatment groups (Tables 1, 2).

**Table 1:** Baseline indices in the UHMV and UHNV groups.

**Baseline indexes**	**Choice of operation**		***P-*value**
	**Neuroendoscope group (*n* = 20)**	**Microscope group (*n* = 21)**	
**Sex (%)**			
Male	11 (55)	8 (38.1)	
Female	9 (45)	13 (61.9)	0.278
Age	55.8 ± 9.4	53.8 ± 12.8	0.65
**Symptom (%)**			
Spinal root pain	11 (55)	9 (42.9)	
Symptoms of spinal cord	8 (40)	10 (47.6)	
Urine or stool incontinence	1 (5)	2 (9.5)	0.719
**Segment (%)**			
Cervical vertebra	5 (25)	3 (14.3)	
Thoracic vertebra	6(30)	11 (52.4)	
Lumbar vertebra	8 (40)	7 (33.3)	
Sacral vertebrae	1 (5)	0 (0)	0.438
**Location (%)**			
Under endorhachis	14 (70)	16 (76.2)	
Dumbbell-shaped	6 (30)	5 (23.8)	0.681
**Intraspinal position (%)**			
Left	10 (50)	9 (42.9)	
Right	8 (40)	8 (38.1)	
Middle	2 (10)	4 (19)	0.833
**Tumor size (mm)**			
Vertical diameters	21.3 ± 8.09	20.0 ± 8.19	0.657
Transverse diameters	16.55 ± 4.27	16.43 ± 6.39	0.957

**Table 2:** Basic characteristics of 41 patients.

	**Age (Year)**	**Gender**	**Diagnosis**	**Surgery**	**Total/Su–btotal resection**	**Preoperative neurological status**	**Postoperative neurological status**	**Bedridden Time (day)**	**Follow-up (Year)**
1	43	Male	T^10^-T^12^Schwannoma	UHMV	Complete	Symptoms of spinal cord	NO	3	1
2	68	Female	C^4^-C^5^Spinal meningioma	UHMV	Complete	Spinal root pain	subcutaneous effusion	6	1
3	56	Male	L^1^-L^2^Spinal meningioma	UHMV	Complete	Spinal root pain	NO	4	1.5
4	53	Female	C^7^-T^2^Schwannoma	UHMV	Residual	Spinal root pain	Limb numbness	3	1
5	51	Male	L^1^-L^3^Schwannoma	UHMV	Complete	Symptoms of spinal cord	NO	4	1
6	48	Male	T^8^-T^9^Spinal meningioma	UHMV	Complete	Symptoms of spinal cord	Limb numbness	3	1
7	50	Female	T^3^-T^4^Schwannoma	UHMV	Complete	Symptoms of spinal cord	NO	3	2
8	64	Female	C^5^-C^7^Schwannoma	UHMV	Complete	Spinal root pain	Limb numbness	4	1
9	77	Male	T^7^-T^9^Schwannoma	UHMV	Complete	Symptoms of spinal cord	NO	6	1
10	34	Female	T^11^-T^12^Spinal meningioma	UHMV	Complete	Spinal root pain	Limb numbness	3	1
11	31	Female	L^2^-L^3^Schwannoma	UHMV	Complete	Symptoms of spinal cord	NO	3	1
12	61	Female	T^10^-T^11^Spinal meningioma	UHMV	Complete	Spinal root pain	NO	4	1.5
13	59	Female	T^10^-L^1^Schwannoma	UHMV	Residual	Symptoms of spinal cord	NO	4	1
14	64	Female	T^6^-T^8^Schwannoma	UHMV	Complete	Spinal root pain	Limb numbness	5	1
15	56	Female	T^12^-L^2^Schwannoma	UHMV	Complete	Spinal root pain	NO	4	1
16	49	Female	C^3^-C^6^Schwannoma	UHMV	Complete	Symptoms of spinal cord	subcutaneous effusion	6	1.5
17	67	Female	T^5^-T^7^Schwannoma	UHMV	Complete	Symptoms of spinal cord	NO	5	1
18	56	Female	L^4^-L^5^Schwannoma	UHMV	Complete	Symptoms of spinal cord	NO	4	1
19	69	Male	T^9^-T^11^Schwannoma	UHMV	Complete	Spinal root pain	Limb numbness	5	1
20	46	Male	L^3^-L^5^Spinal meningioma	UHMV	Complete	Urine incontinence	NO	3	1
21	28	Male	L^4^-S^1^Metastatic tumor	UHMV	Complete	stool incontinence	NO	3	0.5
22	53	Male	C^2^-C^5^Schwannoma	UHNV	Complete	Spinal root pain	Limb numbness	2	1
23	67	Female	L^4^-L^5^Spinal meningioma	UHNV	Complete	Spinal root pain	NO	3	1
24	49	Female	T^5^-T^6^Schwannoma	UHNV	Complete	Symptoms of spinal cord	NO	2	1
25	46	Male	L^2^-L^3^Spinal meningioma	UHNV	Complete	Spinal root pain	NO	2	1
26	50	Male	C^5^-C^7^Spinal meningioma	UHNV	Complete	Spinal root pain	NO	2	1
27	70	Male	T^10^-T^12^Schwannoma	UHNV	Complete	Spinal root pain	Limb numbness	4	1
28	57	Male	L1Schwannoma	UHNV	Complete	Spinal root pain	NO	3	1
29	67	Female	C^4^-C^6^Spinal meningioma	UHNV	Complete	Spinal root pain	NO	3	1
30	63	Male	T^9^-T^10^Spinal meningioma	UHNV	Complete	Symptoms of spinal cord	NO	3	1
31	47	Male	T^7^-T^8^Schwannoma	UHNV	Complete	Symptoms of spinal cord	NO	2	1
32	45	Female	C^4^-C^5^Schwannoma	UHNV	Complete	Spinal root pain	Limb numbness	2	1
33	53	Male	T^12^-L^2^Schwannoma	UHNV	Complete	Symptoms of spinal cord	NO	2	1
34	47	Female	L^3^-L^4^Spinal meningioma	UHNV	Complete	Symptoms of spinal cord	NO	2	1
35	54	Male	C^5^-C^7^Spinal meningioma	UHNV	Complete	Spinal root pain	NO	2	1
36	50	Male	L^3^-L^4^Schwannoma	UHNV	Complete	Symptoms of spinal cord	Limb numbness	2	1
37	60	Male	L^1^-L^2^Spinal meningioma	UHNV	Complete	Spinal root pain	NO	3	1
38	55	Female	T^2^-T^4^Schwannoma	UHNV	Complete	Symptoms of spinal cord	NO	2	1
39	72	Female	L^2^-L^4^Spinal meningioma	UHNV	Complete	Symptoms of spinal cord	NO	4	1
40	53	Female	T^11^-T^12^Spinal meningioma	UHNV	Complete	Spinal root pain	NO	2	1
41	71	Female	S^1^-S^2^Metastatic tumor	UHNV	Complete	Urine incontinence	NO	4	0.5

### Intraoperative Indicators in the Two Treatment Groups

Intraoperative indicators including incision length, operative duration, and intraoperative blood loss were compared between the two groups (Table 3). The surgical incision length was significantly shorter in the UHNV group (5.55 ± 1.29 cm) compared to the UHMV group (8.09 ± 2.43 cm) (*P* < 0.05). There were no differences in operative duration between these groups (225.24 ± 65.07 vs. 214.55 ± 36.23 min), while intraoperative blood loss was significantly reduced in the UHNV group as compared to the UHMV group (95.45 ± 41.56 vs. 172.86 ± 51.20 mL; *P* < 0.05). There were no differences in the vertical and horizontal diameters of the tumor between groups. between groups. (*P*_verticaldiameters_ > 0.05, *P*_horizontaldiameters_ > 0.05).

**Table 3:** Intraoperative indicators in the UHMV and UHNV groups.

	**Incision length (cm)**	**Operation duration (min)**	**Intraoperative blood loss (mL)**
Neuroendoscope group (*n* = 20)	5.55 ± 1.29	214.55 ± 36.23	95.45 ± 41.56
Microscope group(*n* = 21)	8.09 ± 2.43	225.24± 65.07	172.86 ± 51.20
*t*	−3.24	−0.503	−4.31
*p*	0.03	0.619	<0.001

### Indicators of Curative Efficacy in the Two Treatment Groups

Indicators of curative efficacy were then compared between patient groups (Table 4). Postoperative improvement rates were 100% (20/20) in the UHNV group and 95.3% (20/21) in the UHMV group, showing no significant difference (*P* = 1.000). Following surgery, both groups experienced varying degrees of improvement in preoperative symptoms such as nerve radiculopathy, paresthesia, incontinence, and muscle weakness. However, one patient with urinary incontinence in the UHMV group did not experience symptomatic recovery. The total resection rates did not differ significantly between the UHNV and UHMV groups (100 vs. 90.5%, respectively; *P* > 0.05). The UHNV group had lower recurrence rates than the UHMV group (0 *vs*. 14.3%), but the difference was not statistically significant (*P* > 0.05).

**Table 4:** Comparison of baseline efficacy and safety indexes in the UHMV and UHNV groups.

	**Neuroendoscope group (*n* = 20)**	**Microscopic group (*n* =21)**	***P-value***
**Postoperative symptoms (%)**			
Unimproved	0 (0)	1 (4.7)	
Improved	20 (100)	20 (95.3)	
Exacerbation	0 (0)	0 (0)	1
**Pathology (%)**			
Schwannoma	9 (45)	14 (66.7)	
Spinal meningioma	10 (50)	6 (28.6)	
Else	1 (5)	1 (5.7)	0.356
**Complication (%)**			
No	20 (100)	19 (90.5)	
Yes	0 (0)	2 (9.5)	0.488
**Tumor resection (%)**			
Complete	20 (100)	19 (90.5)	
Residual	0 (0)	2 (9.5)	0.157
**Relapse (%)**			
No	20 (100)	18 (85.7)	
Yes	0 (0)	3 (14.3)	0.079
Bedridden Time (day)	2.55 ± 0.76	4.05 ± 1.05	<0.001
Length of stay (LOS) (day)	6.27 ± 0.47	9 ± 1.48	<0.001

### Safety Indicators in the Two Treatment Groups

Next, safety outcomes between patient groups were compared, including complication rates, pathological findings, bedridden time, and length of stay (LOS) (Table 4). There were no reported complications in the UHNV group, while two patients (9.5 percent) from the UHMV group suffered from delayed wound healing due to subcutaneous effusion. However, the difference was not statistically significant (*P* > 0.05).

Moreover, the results showed that the bedridden time of the UHNV group was significantly shorter by 1.3 days (*P* < 0.001). Similarly, the mean length of stay (LOS) in the UHNV group (6.27 ± 0.47 d) was significantly shorter than in the UHMV group (9 ± 1.48 d) (*P* < 0.001). Follow-up MRI and CT radiographs of these patients postoperatively revealed no evidence of spinal instability, spinal stenosis, or recurrence. Kanrnofsky Performation Scale (KPS) and Functional Independence Measurement Scale (FIMTM) were used to assess patients' quality of life after 1 year. It was determined that all patients were able to live and work normally.

**Figure 3:**
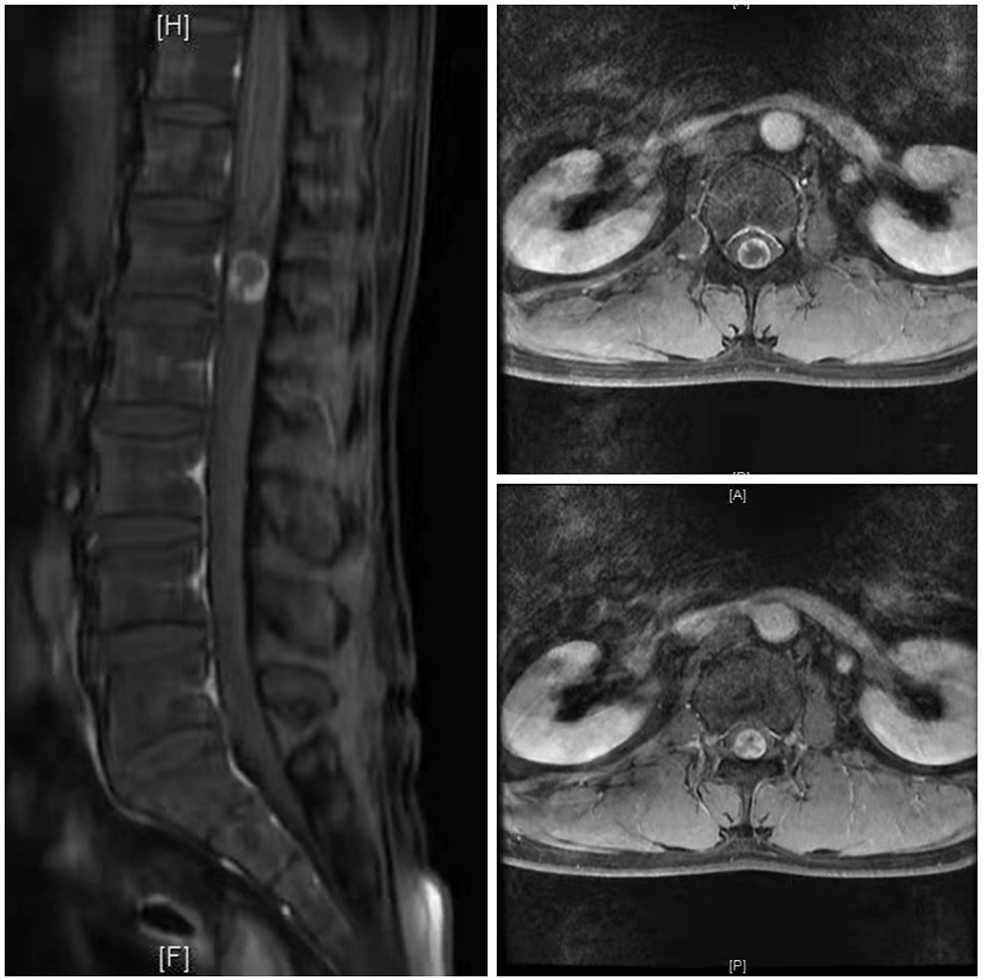
Preoperative MRI of the case 1.

**Figure 4:**
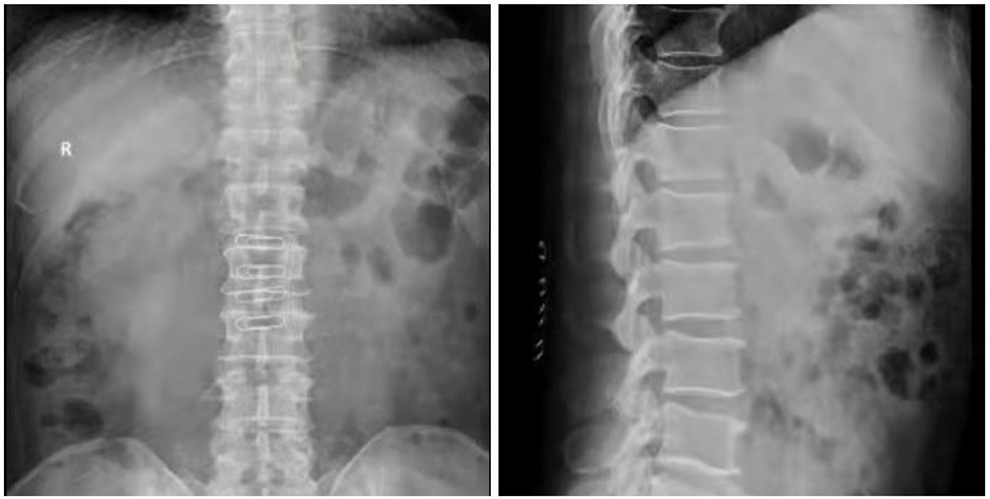
Position the cone preoperatively of the case 1.

**Figure 5:**
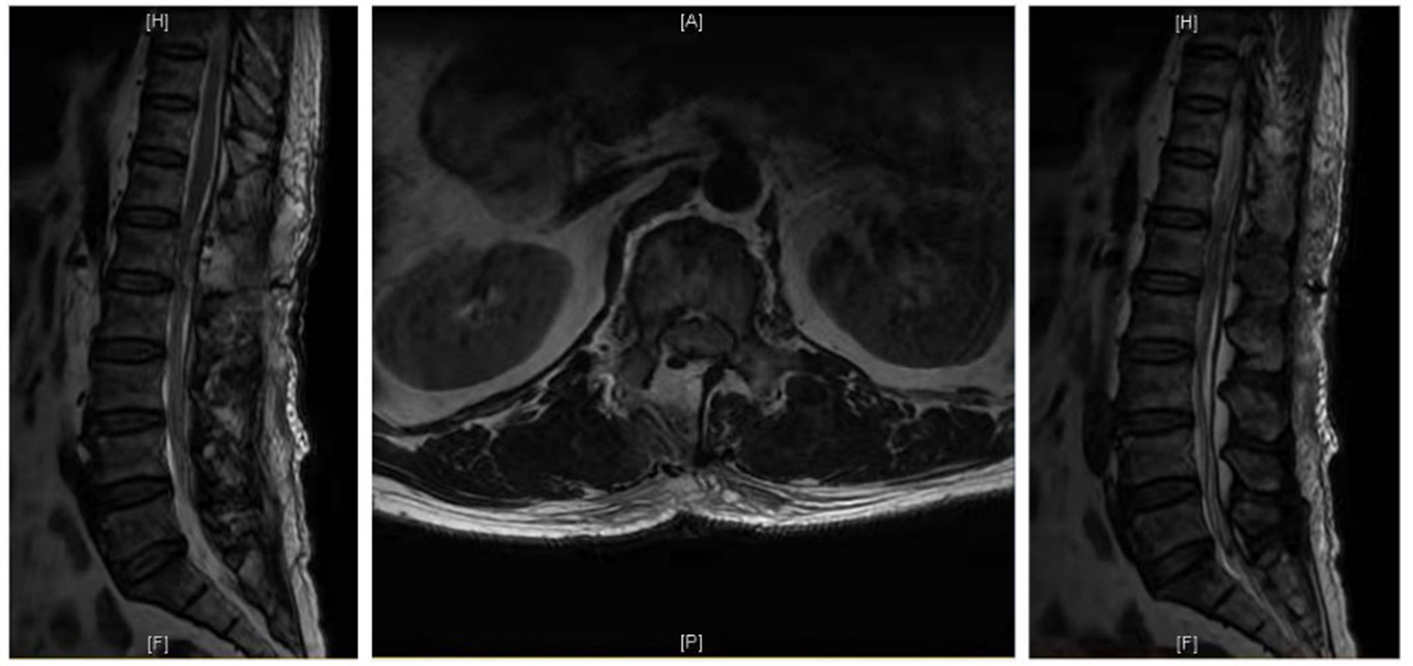
Postoperative MRI of the case 1.

**Figure 6:**
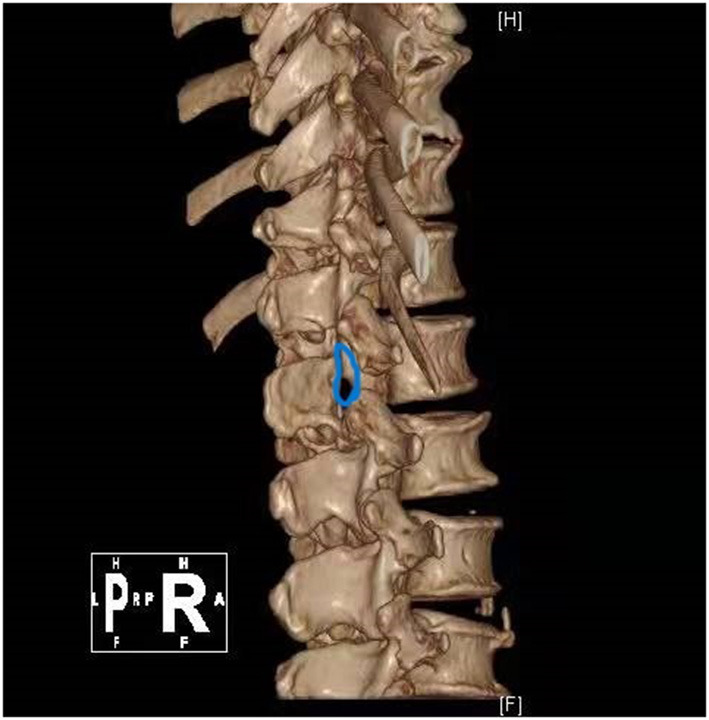
Postoperative 3D CT reconstruction of the case 1.

Case 1: A 57-year-old male complaining of pain in his right lower back and leg for one month. Contrast-enhanced magnetic resonance imaging of the spine revealed a lesion posterior to the spinal cord at the L1 level (Figure 3). The patient underwent endoscopic surgery following preoperative positioning (Figure 4). A small amount of bone was removed relative to the size and location of the tumor to enlarge the corresponding laminal space and expose the tumor. An MRI performed postoperatively revealed complete resection of the tumor (Figure 5). Three-dimensional computed tomography confirmed good spinal stability (Figure 6). The pathological examination revealed the presence of schwannoma, and the patient experienced no pain at the follow-up visit.

## Discussion

### Laminectomy

Total laminectomy, as performed by Gowers et al. in 1888, was the most fundamental and classic surgical technique for intraspinal tumors, from which all subsequent surgical techniques evolved (10). The laminectomy procedure involves the exposure of the tumor via surgical resection of the supraspinous ligament, interspinous ligament, spinous process, and entire vertebral lamina. This approach enables full lesion visualization, thus providing a clear operative field for surgical resection. Thus, total laminectomy under a microscope is appropriate for patients with spinal stenosis and multi-segment massive tumors in the spinal canal, as it safely removes the tumor while exerting minimal traction on the spinal cord and nerve roots (11). However, patients need to stay in bed for a long time after surgery, which may lead to serious complications. Furthermore, severe loss of posterior column structures such as the laminar ligament, loss of bony structural support for diseased segments, and loss of paravertebral muscle attachment points may result in long-term spinal deformity. According to Onyia CU et al., there is a 60% chance that a second operation will be required due to spinal instability following total laminectomy (12). As a result, some studies recommend performing internal fixation with a screw and rod fixation system when using the total laminectomy approach. While this procedure effectively prevents postoperative spinal instability, it severely restricts the range of motion of the corresponding segment and may result in accelerated degeneration of adjacent segments. Additionally, screw implants carry risks, including injury to the spinal cord and nerve roots during implantation and long-term risks, such as neurological symptoms associated with screw loosening and fracture. Because of the direct contact between the spinal canal structure and surrounding soft tissues, spinal dura mater and nerve root adhesions are quite common. According to Raffaeli et al., scar tissues surrounding the nerve root may impair the nerve root's blood supply and nutrition, resulting in chronic nerve root pain following surgery (13).

### Hemilaminectomy

In 1908, Taylor AS et al. came up with the hemilaminectomy technique, thereby launching the era of minimally invasive spine surgery (14). This technique required only one side of the lamina to be removed, thereby preserving the midline spinal ligament complex and contralateral muscles necessary for spinal stability. The posterior ligament complex (such as the interspinous ligament) serves as a point of attachment for the muscle, and its preservation allows for paravertebral muscle reconstruction along the natural plane, increasing the likelihood of muscle recovery and minimizing the risk of muscle atrophy and subsequent kyphosis (15). Perez-Cruet et al. (16) previously found that an increasing number of experimental and clinical studies had established a strong correlation between vertebral column stability and the vertebral lamina's decompression degree. As a result, in clinical settings and evidence-based studies, the UHMV procedure has largely superseded total laminectomy (5, 17–20). The disadvantage of hemilaminectomy is that the surgical area between the spinous process and the ipsilateral facet joint is small, and microscope-based visualization is limited by a small depth of field, tubular vision, and inadequate illumination of deeper sites. As a result, UHMV is prone to insufficient tumor resection and unintentional spinal cord and nerve root injury. The advent of neuroendoscopic hemilaminectomy by Endo T et al. and Burkhardt BW et al. has revolutionized how hemilaminectomy is performed, with an increasing number of people believing that microscopic visualization will gradually be replaced (21, 22).

### Split Laminotomy

Bognár et al. (23) reported the first successful laminectomy in six pediatric patients. The benefits of this procedure include symmetrical reconstruction of the posterior column of the spine and a low risk of bleeding due to the sparse veins in the dorsal midline epidural space. However, its disadvantages are also quite apparent. (1) After laminectomy, the horizontal width exposed is only about 1.5 cm, the operative field is constrained, and duraplasty is very challenging. (2) Occasionally, the spinous process can fracture during the separation process and could also fracture under traction (24).

### Other (Neuroendoscopic Transforaminal and Interlaminar Surgeries)

At present, endoscopy is widely used to treat degenerative spinal diseases, particularly lumbar disc herniation and lumbar spinal stenosis. Due to technological advancements and increased surgical experience, endoscopic spine surgery indications have expanded considerably. Senturk et al. (25) previously described a translaminar approach for complete endoscopic removal of a lumbar intradural extramedullary tumor. Moreover, Tsai et al., Ying et al., and Zhu et al. (26–28) also supported the use of the interlaminar approach to remove intraspinal tumors. The main benefit of neuroendoscopic transforaminal and interlaminar resection is the ability to achieve complete tumor resection with minimal bone removal with improved spinal mobility and stability preservation. Among the other advantages are decreased intraoperative blood loss and postoperative pain, as well as a shorter hospital stay. Although there are few studies and controversy surrounding these surgical procedures, with the development of endoscopic instruments in recent years, endoscopic technology will become an effective alternative for treating spinal tumors surgically.

Herein, we collected important surgical information on spinal cord meningiomas and schwannomas in the past 10 years and summarized the representative articles (Table 5).

**Table 5:** Surgical approachs for patients with intraspinal tumors.

**Researcher**	**Number**		**operation**		**Surgical tool**		**Pathology**	**IEM**
	**T**	**C**	**T**	**C**	**T**	**C**		
Maduri et al. (29)	13		Tubular retractor		M		IDEM	NO
Stefini et al. (30)	11		Navigation		M		IDEM	NO
Mannion et al. (31)	11		HM		M		IDEM	NO
Li et al. (32)	20	12	LM	LM+TS	M		Dumbbell tumor	NO
Li et al. (33)	50	52	Keyhole	LM	M		IDEM	YES
Zhu et al. (26)	3	15	Interlaminar		N+M	M	IDEM	NO
Wong et al. (34)	27	18	HM	LM	M		IDEM	NO
Li et al. (35)	2		HM+TTIF		M		Dumbbell tumor	NO
Archavlis et al. (36)	7		HM		N		IDEM	NO
Dhandapani et al. (37)	16		HM		N		IDEM	NO
Telfeian et al. (38)	1		Transforaminal		N		IDEM	NO
Xie et al. (39)	11		cannula		N		IDEM	NO
Formo et al. (40)	83		HM		M		IDEM	NO
Balasubramanian et al. (41)	41		Keyhole		M		IDEM	NO
Soriano Sánchez et al. (42)	13		NET		M		IDEM	NO
Thavara et al. (43)	12		Tubular retractor		M		IDEM	NO
Pompili et al. (44)	97		HM		M		IDEM	NO
Nzokou et al. (45)	13		Tubular retractor		M		IDEM	NO
Dobran et al. (46)	20	20	HM	LM	M		IDEM	NO
Rösler et al. (47)	37		HM		N		IDEM	NO
Parihar et al. (48)	14		HM		Exoscope+N		IDEM	NO
Teo et al. (49)	8		HM		Exoscope		IDEM	NO
Pojskić et al. (50)	1		HM + thoracotomy		M + thoracoscopic		Dumbbell tumor	NO
Yan et al. (51)	20		LM		N		IDEM	Yes
Yang et al. (52)	7		Interlaminar + thoracotomy		N + thoracoscopic		Dumbbell tumor	NO
Carl et al. (53)	10		LM		Microscope–based AR		IDEM	NO
Iacoangeli et al. (54)	30		HM		M		IDEM	Yes

*IDEM, intradural extramedullary; HM, hemilaminectomy; LM, laminectomy; TS, thoracoscopic surgery; M, microscope; N, neuroendoscopy; TTIF, unilateral transforaminal thoracic intervertebral fusion; NET, Non-Expansile Tubular; AR, Augmented reality*.

In this study, most patients undergoing UHMV exhibited satisfactory tumor resection and postoperative improvements in clinical symptoms, in line with prior reports (8). Meanwhile, the UHNV group had favorable outcomes, and this approach was associated with benefits such as decreased blood loss, tissue injury, hospitalization duration, length of stay (LOS), and postoperative complication rates. Parihar et al. previously evaluated the outcomes of 18 patients who underwent UHNV and determined that this technique was a safe and effective alternative to the UHMV approach (55). Di et al. similarly observed reductions in soft tissue injury, blood loss, incision length, postoperative pain, and hospitalization duration in patients who underwent the UHNV procedure (56). Besides, Ren et al. reported that UHNV was an effective minimally invasive procedure that reduced the presence of residual tumors in treated patients (57). Overall, small bony fenestrations or enlarged interlaminar corridors offer a similar effect to tubular retractors, and using endoscopy with “tubular retractors” provides a better view than “tubular” microsurgery.

**Figure 7:**
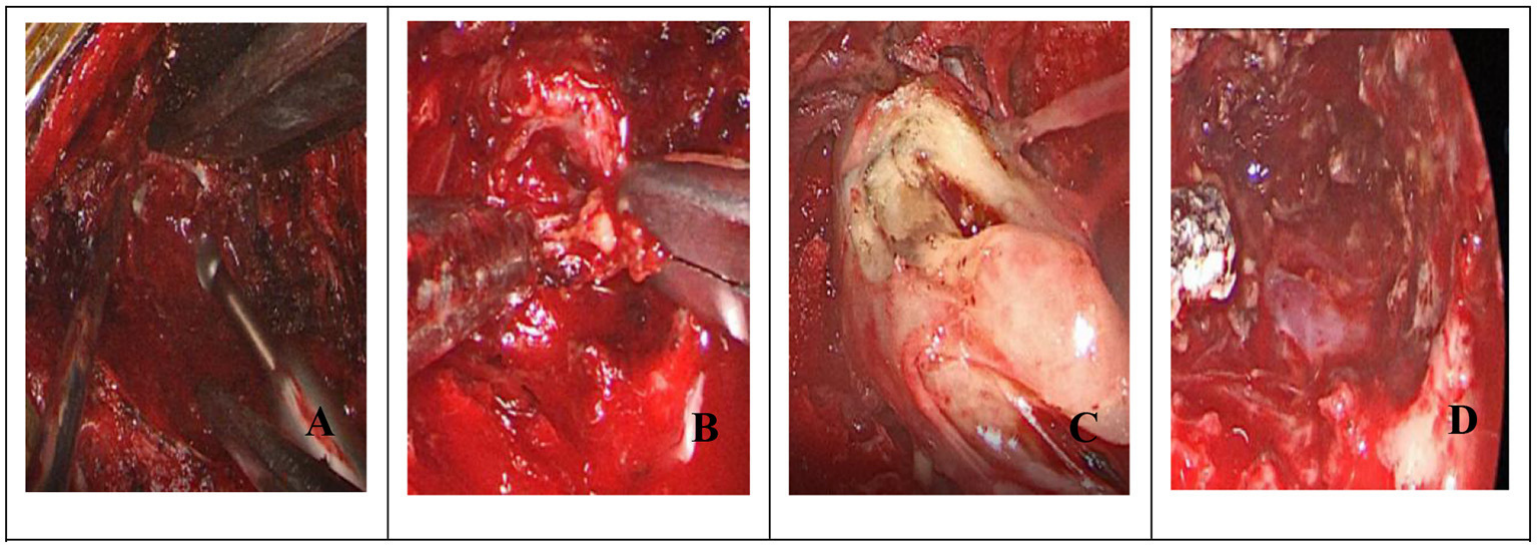
(Dumbbell): **(A)** Endoscopic removal of hemilamellae; **(B)** Endoscopic intratumoral resection; **(C)** After shrinking the tumor to create a channel, carefully separate the tumor from upper and lower levels; **(D)** Tumor removal following endoscopic electrocoagulation.

In light of our findings and prior studies, we believe that the UHNV procedure has several significant advantages over the UHMV procedure. Among them are the following:

(1) For tumors of comparable size (diameter, length, and location), surgical incisions were typically 3–4 cm longer in the UHMV group than in the UHNV group. Due to the larger incision and associated scar tissue development, these larger surgical incisions are likely to increase the incidence of postoperative complications such as pain, infection, myasthenia, and cerebrospinal fluid leakage (58).
(2) The UHNV procedure can minimize soft tissue separation and excessive bone removal, thereby avoiding extensive muscle stripping and lowering postoperative pain, bleeding, and spinal instability rates (59).
(3) Neuroendoscopic visualization enables a more reliable panoramic view of the operating field, allowing for more detailed examination and illumination in deep tissue sites (60). As the operation progresses, the neuroendoscope can be continuously adjusted to meet changing operative requirements, allowing for more precise tumor separation and resection. For dumbbell-shaped intraspinal tumors primarily located outside the spinal canal or for other large tumors, an initial tumor portion can be removed to create a channel, followed by careful separation of the upper and lower tumor edges and segmental tumor resection (Figure 7). Overall, our findings corroborate with those of Barrenechea et al. (61) and offer further insight into this field.
(4) Using an angled lens eliminates blind spots within the visual field during surgery. The use of a flexible neuroendoscope that enables “round-angle observation” can ensure complete tumor resection and visualization of the spinal canal from multiple angles and directions while minimizing iatrogenic damage to the spinal cord, nerve roots, or blood vessels, thereby lowering the incidence of postoperative complications. This advantage is especially advantageous when tumors are located primarily in the midline and have a large vertical diameter, when tumors are closely adherent to the spinal cord or nerve roots, or when tumors are located ventrally or laterally.
(5) The UHNV approach is minimally invasive, allowing for a more rapid postoperative recovery, a shorter length of stay, lower hospitalization costs, and more efficient use of medical resources. Singh et al. (62) also confirmed that the UHNV approach is superior due to its accessibility, mobility, illumination, and visualization advantages.

There are certain limitations associated with the UHNV approach. First, the neuroendoscopic approach imposes a high burden on the operator, who must be familiar with correct neuroendoscopic surgical protocols. Additionally, this approach necessitates improved hand-eye coordination, spatial positioning, and additional training on cadavers before clinical translation (63).

Nevertheless, this study has some limitations. Firstly, this was a non-randomized cohort study which might cause selection bias. Secondly, due to the rarity of intramedullary and spinal cord malignancies, those patients were excluded from this study. As a result, additional research is warranted to determine the safety and efficacy of UHNV in treating intramedullary and spinal cord malignancies. Furthermore, this was a relatively small study with a short-term follow-up period, and additional large-scale studies with a longer follow-up period will be needed to confirm and expand upon the results of this study.

## Conclusions

The resection of intradural extramedullary tumors using the UHNV technique is a safe and effective alternative to the UHMV approach while also minimizing surgical injury.

## Data Availability Statement

The raw data supporting the conclusions of this article will be made available by the authors, without undue reservation.

## Ethics Statement

Ethical review and approval was not required for the study on human participants in accordance with the local legislation and institutional requirements. Written informed consent for participation was not required for this study in accordance with the national legislation and the institutional requirements.

## Author Contributions

WZ: conception and design and manuscript writing. CW: administrative support. WZ, HJ, and SH: provision of study materials or patients. WZ, YZ, and HJ: collection and assembly of data. WZ, BY, and HW: data analysis and interpretation. All authors approved the final manuscript.

## Conflict of Interest

The authors declare that the research was conducted in the absence of any commercial or financial relationships that could be construed as a potential conflict of interest.

## Publisher's Note

All claims expressed in this article are solely those of the authors and do not necessarily represent those of their affiliated organizations, or those of the publisher, the editors and the reviewers. Any product that may be evaluated in this article, or claim that may be made by its manufacturer, is not guaranteed or endorsed by the publisher.

## Supplementary Material

Supplementary Video 1 Endoscopically, we looked for the location of the tumor and carefully isolated the peripheral nerves.

Supplementary Video 2 Under endoscopy, we isolated the tumor and removed part of it to expand the surgical space.

Supplementary Video 3 Endoscopically, we isolated the parent nerve while protecting the nerve.

Supplementary Video 4 An initial tumor portion can be removed to create a channel, and remaining tumor segmental resection.

Supplementary Video 5 Complete tumor resection, tumor cavity hemostasis.

Supplementary Video 6 The corresponding vertebral laminae were partially removed under neuroendoscope according to location and size, and basal range of tumor, and the window size is suitable for the operation of two instruments such as an aspirator and a detacher under endoscopic visualization.

Supplementary Video 7 After careful hemostasis, the endorhachis was cut longitudinally.

Supplementary Video 8 The endorhachis was cut longitudinally, and parts of the tumor can be removed to create a channel.
